# Array comparative genomic hybridization identifies high level of PI3K/Akt/mTOR pathway alterations in anal cancer recurrences

**DOI:** 10.1002/cam4.1533

**Published:** 2018-05-26

**Authors:** Wulfran Cacheux, Petros Tsantoulis, Adrien Briaux, Sophie Vacher, Pascale Mariani, Marion Richard‐Molard, Bruno Buecher, Sophie Richon, Emmanuelle Jeannot, Julien Lazartigues, Etienne Rouleau, Odette Mariani, Elsy El Alam, Jérôme Cros, Sergio Roman‐Roman, Emmanuel Mitry, Elodie Girard, Virginie Dangles‐Marie, Astrid Lièvre, Ivan Bièche

**Affiliations:** ^1^ Département d’oncologie médicale Institut Curie, Ensemble hospitalier Hôpital René Huguenin Saint‐Cloud France; ^2^ Unité de pharmacogénomique Département de génétique Institut Curie, Ensemble hospitalier Paris Cedex 05 France; ^3^ Centre d’oncologie Hôpitaux universitaires de Genève Genève Switzerland; ^4^ Département de chirurgie oncologique Institut Curie, Ensemble hospitalier Paris Cedex 05 France; ^5^ Département de radio‐oncologie Institut Curie, Ensemble hospitalier Hôpital René Huguenin Saint‐Cloud France; ^6^ Département d’oncologie médicale Institut Curie, Ensemble hospitalier Paris Cedex 05 France; ^7^ Centre de recherche Institut Curie UMR144 Paris Cedex 05 France; ^8^ Département d’anatomopathologie Institut Curie, Ensemble hospitalier Paris Cedex 05 France; ^9^ Département d’anatomopathologie Institut Curie, Ensemble hospitalier Hôpital René Huguenin Saint‐Cloud France; ^10^ Recherche translationnelle Centre de recherche Institut Curie Paris Cedex 05 France; ^11^ Département de bio‐informatiques Centre de recherche Institut Curie Paris Cedex 05 France; ^12^ IFR71 Faculté des sciences biologique et pharmacologiques Université Paris Descartes Sorbonne Paris Cité Paris France; ^13^ Département de gastroentérologie Hôpital universitaire de Rennes Université de Rennes 1 Rennes France

**Keywords:** anal squamous cell carcinoma, array comparative genomic hybridization, copy number alterations, PI3K/Akt/mTOR signaling pathway, somatic mutations

## Abstract

Genomic alterations of anal squamous cell carcinoma (ASCC) remain poorly understood due to the rarity of this tumor. Array comparative genomic hybridization and targeted gene sequencing were performed in 49 cases of ASCC. The most frequently altered regions (with a frequency greater than 25%) were 10 deleted regions (2q35, 2q36.3, 3p21.2, 4p16.3, 4p31.21, 7q36.1, 8p23.3, 10q23.2, 11q22.3, and 13q14.11) and 8 gained regions (1p36.33, 1q21.1, 3q26.32, 5p15.33, 8q24.3, 9q34.3, 16p13.3, and 19p13.3). The most frequent minimal regions of deletion (55%) encompassed the 11q22.3 region containing *ATM*, while the most frequent minimal regions of gain (57%) encompassed the 3q26.32 region containing *PIK3CA*. Recurrent homozygous deletions were observed for 5 loci (ie, *TGFR2* in 4 cases), and recurrent focal amplifications were observed for 8 loci (ie, *DDR2* and *CCND1* in 3 cases, respectively). Several of the focal amplified genes are targets for specific therapies. Integrated analysis showed that the PI3K/Akt/mTOR signaling pathway was the pathway most extensively affected, particularly in recurrences compared to treatment‐naive tumors (64% vs 30%; *P *=* *.017). In patients with ASCC recurrences, poor overall survival (OS) was significantly correlated with a large number of altered regions (*P *=* *.024). These findings provide insight into the somatic genomic alterations in ASCC and highlight the key role of the druggable PI3K/Akt/mTOR signaling pathway.

## 1 INTRODUCTION

Anal squamous cell carcinoma (ASCC) is a rare tumor, but its incidence has been increasing over the past 2 decades.^1^, ^2^, ^3^ This cancer is closely related to human papillomavirus (HPV) infection.^4^ Most patients are diagnosed with locally advanced disease, for which the standard of care is chemoradiotherapy (CRT).^5^ High complete response rates are obtained, but 20% of patients are nonresponders or relapse within the first 3 years after treatment. Salvage abdominoperineal resection (APR) is the standard treatment for local failure or recurrence after CRT, but 30% to 60% of operated patients subsequently experience locoregional and/or metastatic recurrence.^6^, ^7^ Very few treatments with very limited efficacy are available for these patients with inoperable locally advanced or metastatic disease. A better understanding of the molecular markers involved in anal carcinogenesis is necessary in order to identify new therapeutic targets as well as prognostic and predictive biomarkers. In comparison with other squamous cell carcinomas and HPV‐related cancers, the molecular landscape of ASCC is currently not well characterized and few genomic studies are available.^8^, ^9^, ^10^ Only limited and old data are available concerning the recurrent pattern of chromosomal aberrations in ASCC.^11^, ^12^ Only one study was based on a comparative genomic hybridization (CGH) approach, but concerned a cohort of 35 cases of anal intra‐epithelial neoplasia.^13^ In this study, we present the results of array‐CGH analysis of 49 ASCC patients with comparison of genomic profiles between treatment‐naive tumors and recurrences.

## 2 MATERIALS AND METHODS

### 2.1 Sample collection

Forty‐nine tumor samples from 49 patients with ASCC (ie, no paired samples from same patients) treated between 1992 and 2015 at the Institut Curie Hospital were retrospectively analyzed. All biopsy tissues were residual specimens and macrodissected to achieve maximum tumor purity. A fresh frozen tumor sample was considered suitable for the study when the proportion of tumor cells exceeded 70%. This retrospective study was reviewed and approved by the Institut Curie Ethics Committee (No. A10‐024). According to French regulations, patients were informed about the research performed on the biological specimens obtained during their treatment and did not express any opposition. Clinical and laboratory data were collected for each patient. Disease staging was based on the 7th revised edition (2010) of the American Joint Committee on Cancer (AJCC) staging of anus cancer. Fifteen samples of adjacent normal anal squamous cell tissue from patients with ASCC were used as sources of normal RNA for RT‐qPCR. Tissues samples were stored at −70°C until DNA and RNA extractions.

### 2.2 Genomic DNA extraction

The Qiagen DNeasy Tissue kit and the protocols for fresh frozen ASCC tissues were used. DNA was purified by column purification with a filter membrane and stored at −20°C before use.

### 2.3 Total RNA extraction

Total RNA was extracted from fresh frozen ASCC and normal anal squamous cell tissues by the acid‐phenol guanidium method. The quantity of RNA was assessed using an ND‐1000 NanoDrop Spectrophotometer with its corresponding software (Thermo Fisher Scientific Inc., Wilmington, DE). RNA quality was determined by electrophoresis on agarose gel with ethidium bromide staining. The 18S and 28S RNA bands were visualized under ultraviolet light. Total RNA was stored at −20°C before use.

### 2.4 HPV genotyping

HPV status was assessed in the Institut Curie Pathology Department. Total DNA, isolated from formalin‐fixed tissue blocks, was used for HPV typing. Real‐time PCR using Sybr^®^Green and specific primers for HPV16 and 18 was performed on a 7900HT Fast Real‐Time PCR System (Applied Biosystems, Foster City, CA).

### 2.5 Mutation assessment

HRM primers for screening mutations were designed for *KRAS* (exons 2, 3, and 4), *BRAF* (exon 15), *PIK3CA* (exons 9 and 20), and *TP53* (exons 5‐8). PCR for HRM analysis was performed on a 384‐well plate in the presence of the fluorescent DNA intercalating dye, LC green (Idaho Technology) in a LightCycler480^®^ (Roche). HRM analysis was performed with Genescan software (Roche). All samples were plotted according to their melting profiles on the differential plot graph. All samples were sequenced using Sanger sequencing approach, whenever an abnormal HRM curve was suspected. The nucleotide sequences of the oligonucleotide primers for the genes examined are listed in Table S1.

### 2.6 Array‐CGH

ASCCs were tested using a 400K human genome CGH microarray. Array‐CGH experiments were carried out using standard Agilent protocols (Agilent Technologies, Santa Clara, CA). Commercial human genomic DNA (Agilent Technologies) was used as diploid reference. Briefly, 1‐1.5 μg of reference DNA and the same amounts of patient tumor DNA were digested with Alu1 and Rsa1 (Promega, Madison, WI, USA). The digested reference DNA fragments were labeled with cyanine 3‐dUTP, and tumor DNA was labeled with cyanine 5‐dUTP (Agilent Technologies). After cleanup, labeled reference and tumor DNA were mixed as probes and hybridized onto an Agilent 400K human genome CGH microarray (Agilent Technologies) for 40 hours. Washing, scanning, and data extraction procedures were carried out according to standard protocols. Data were extracted using Feature Extraction software (v11.1), and normalized data were analyzed and visualized by Agilent Cytogenomics Edition 2.9.2.4 (Agilent Technologies). The aberration detection module 2 (ADM‐2) with threshold 6 was used to calculate copy number alterations (CNAs). Five‐probe 0.20_log2 filter was used for aberration evaluation, given an average genomic resolution of 7 Kb. DGV database (hg19) was used for elimination of the common copy number polymorphism regions from the dataset. Cytogenomics Edition 2.9.2.4 (Agilent Technologies) was used to calculate the log2 ratio for each probe and to identify genomic aberrations. The mean log2 ratio of all probes in a chromosome region between 0.20 and 1.0 was classified as genomic gain, more than 1.0 (with a size <10 Mb) as focal amplification, less than −0.30 as heterozygous deletion, and less than −1.0 (with a size <5 Mb) as homozygous deletion.

### 2.7 RT‐qPCR

The theoretical and practical aspects of RT‐qPCR have been previously described in detail.^14^ The precise amount of total RNA added to each reaction mix (based on optical density) and its quality (ie, lack of extensive degradation) are both difficult to assess. Transcripts of an endogenous RNA control gene involved in cellular metabolic pathway, namely *TBP* (Genbank accession NM_003194),^15^ which encodes the TATA box‐binding protein (a component of the DNA‐binding protein complex TFIID), were therefore also quantified. Each sample was normalized on the basis of its *TBP* content. Results, expressed as N‐fold differences in target gene expression relative to the *TBP* gene and termed “N*target*,” were determined as N*target* = 2^ΔCtsample^, where the Δ*Ct* value of the sample was determined by subtracting the *Ct* value of the target gene from the *Ct* value of the *TBP* gene. The N*target* values of the samples were subsequently normalized so that the median of the 15 normal anal squamous cell tissue N*target* values was 1. cDNA synthesis and PCR conditions were as previously described.^14^ Primers for *TBP* and the target genes were designed with the assistance of Oligo 6.0 software (National Biosciences, Plymouth, MN). To avoid amplification of contaminating genomic DNA, 1 of the 2 primers was placed at the junction between 2 exons or on between 2 different exons. Agarose gel electrophoresis was used to verify the specificity of PCR amplicons. The nucleotide sequences of the oligonucleotide primers for the selected genes are listed in Table S2.

### 2.8 Statistical analysis

Correlations between molecular parameters (at the RNA or/and DNA level), and clinical, biological, and pathological parameters, were identified using nonparametric tests, namely Chi‐square or Fisher’s exact test (correlation between 2 qualitative parameters), and Kruskal‐Wallis test (correlation between 1 qualitative parameter and 1 quantitative parameter). OS was defined as the interval from the first day of RT or CRT to death from any cause. In order to assess the efficacy of a molecular marker (number of altered regions and fraction of genome altered) to discriminate between 2 populations (alive/deceased patients) in the absence of an arbitrary cutoff value, data were summarized in a ROC (receiver operating characteristic) curve.^16^ The area under curve (AUC) was calculated as a single measure to discriminate efficacy. Survival distributions were estimated by the Kaplan‐Meier method, and the significance of differences between survival rates was ascertained with the log‐rank test. For all statistical tests, differences were considered significant at *P *<* *.05.

## 3 RESULTS

### 3.1 Patient and tumor characteristics

A total of 49 ASCC samples from 49 patients treated in our institution were included and analyzed for CNA and *KRAS, BRAF, PIK3CA,* and *TP53* mutations. Tumor characteristics in the total population according to the treatment‐naive or recurrence status of the samples are summarized in Table S3. Twenty‐seven tumors were treatment‐naive and 22 were samples from recurrence after initial RT or CRT. A total of 46 tumors (93.9%) were HPV‐positive, including 43 tumors (87.5%) with HPV16 infection. Only 4 patients (8.2%) had HIV infection, and all presented concomitant HPV infection. The study population comprised 38 females and 11 males. Eight patients were treated by first‐line surgery: exclusive surgery (n = 4) and surgery followed by RT (n = 3) or CRT (n = 1). Twelve patients were treated by first‐line RT and 29 by first‐line CRT. The median follow‐up of the 49 patients was 46.2 months (range: 9.8 to 278 months). Eight of the 27 treatment‐naive patients relapsed after the initial diagnosis.

### 3.2 Whole‐genome array‐CGH profiles

**Figure 1:**
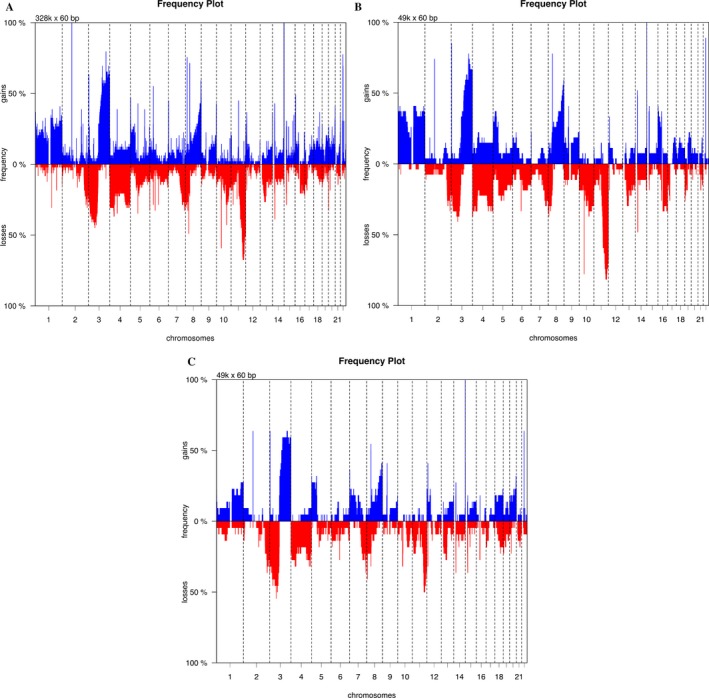
Frequency of chromosomal alterations using array‐CGH in ASCC tumors (*x‐*axis:chromosomes; *y*‐axis:frequency (in percentages) of copy number gains (blue) and losses (red) in the total population of 49 ASCCs (A), in the group of 27 treatment‐naive tumors (B) and in the group of 22 recurrences (C))

Array‐CGH profiles of the 49 ASCCs are represented in Figure 1A. Gains and deletion metrics for each sample are listed in Table S4. The first genomic parameter corresponds to the number of distinct identified CGH segments reflecting the number of break points within the tumor genome. This parameter ranged from 68 to 550, with a median of 137. The second genomic parameter corresponds to the fraction (percentage) of the altered genome. This parameter ranged from 0.96% to 51.94%, with a median of 17.82%.

### 3.3 Heterozygous and homozygous deletions

The most significantly frequent minimal regions of heterozygous deletion with a frequency greater than 25% were located at loci 2q35 (27%), 2q36.3 (29%), 3p21.2 (39%), 4p16.3 (29%), 4p31.21 (27%), 7q36.1 (27%), 8p23.3 (41%), 10q23.2 (27%), 11q22.3 (55%), and 13q14.11 (22%) (Table 1A; Figure 1A). The most common frequent minimal region of heterozygous deletion in 55% of ASCCs encompassed the 8.4‐Mb region containing *ATM* (Figure S1). Other common regions of heterozygous genomic deletion containing well‐known tumor suppressor genes were located at 3p21.2 (*BAP1, PBRM1,* and *FHIT*), 10q23.2 (*PTEN*, that also showed 2 homozygous deletions; Figure S2), and 13q14.11 (*RB1*) (Table 1A). Expression of *ATM* and *PTEN* located in 2 of the smallest common CNAs of interest was then screened for 41 of the 49 ASCC using RT‐qPCR to assess correlations between copy number alterations and mRNA expressions in ASCC tumors. *ATM* and *PTEN* were significantly underexpressed in ASCCs with heterozygous/homozygous deletions, compared to diploid tumors (*P *=* *.006 and *P *=* *.02, respectively; Figures S1 and S2).

**Table 1:** Frequencies (greater than 20%) of loss/deletion (A) and gain/amplification (B) for each chromosomal arm

(A) Loss and deletion									
Chromosomal arm	Locus	Maximal loss and deletion frequency	Genomic position	Common altered genomic region (pb)	Number of genes	Candidate cancer genes	Loss and deletion frequency in naive ASCC (n = 27)	Loss and deletion frequency in recurrent ASCC (n = 22)	*P*‐value naive vs recurrent ASCC (chi‐square test)
2q	2q35	26.53	220197899‐225875177	5677578	30	*PAX3, CUL3*	18.52	36.36	.16 (NS)
2q	2q36.3	28.57	230579286‐233243243	2663957	37	*TRIP12*	29.63	31.82	.87 (NS)
3p	3p21.2	38.78	50712594‐74311719	23599125	167	*BAP1, PBRM1, FHIT*	40.74	54.55	.34 (NS)
4p	4p16.3	28.57	1400230‐44018877	42618647	247	*WHSC1*	29.63	22.73	.59 (NS)
4q	4q31.21	26.53	145659881‐162305043	16645162	78	*FBXW7*	29.63	18.18	.35 (NS)
7q	7q36.1	26.53	151217010‐152133979	916969	5	*KMT2C*	22.22	31.82	.45 (NS)
8p	8p23.3	40.81	419875‐29952921	29533046	266	*NKX3‐1, NEFL, DUSP4*	25.93	18.18	.76 (NS)
10q	10q23.2	26.53	89625664‐89722948	97284	1	*PTEN*	33.33	18.18	.23 (NS)
11q	11q22.3	55.10	107197072‐115631345	8434273	78	*ATM*	62.96	45.45	.22 (NS)
13q	13q14.11	22.45	41837713‐50623108	8785395	85	*RB1*	22.22	22.73	.76 (NS)
Significant (or trending toward) differences between treatment‐naive and recurrent ASCC									
11q	11q14.2	30.61	85631063‐89867817	4236754	28	*EED*	48.14	9.09	.0032
16q	16q11.2	20.41	34990995‐90142338	55151343	475	*CYLD, CBFB, CTCF, CDH1, WWOX*	29.63	4.55	.059 (NS)

(B) Gain and amplification									
Chromosomal arm	Locus	Maximal gain and amplification frequency	Genomic position	Common altered genomic region (pb)	Number of genes	Candidate cancer genes	Gain and amplification frequency in naive ASCC (n = 27)	Gain and amplification frequency in recurrent ASCC (n = 22)	*P*‐value naive vs recurrent ASCC (chi‐square test)
1p	1p36.33	36.73	855072‐1447522	592450	42	*TNFRSF18, TNFRSF4*	33.33	40.91	.58 (NS)
1q	1q21.1	28.57	148951595‐149871154	919559	18	—	29.63	27.27	.86 (NS)
3q	3q26.32	57.14	160788817‐197807542	37018725	284	*TERC, PIK3CA*	59.26	54.55	.75 (NS)
5p	5p15.33	28.57	190087‐535954	345867	11	*AHRR*	22.22	36.36	.28 (NS)
8q	8q24.3	38.78	144442147‐145754562	1312415	71	—	40.74	36.36	.75 (NS)
9q	9q34.3	32.65	138853226‐140201345	1348119	79	*NOTCH1*	33.33	31.82	.91 (NS)
16p	16p13.3	40.82	333003‐903634	570631	37	—	40.74	18.18	.088 (NS)
19p	19p13.3	26.53	474983‐685520	210537	10	*FGFR2*	33.33	18.18	.23 (NS)
Significant differences between treatment‐naive and recurrent ASCC									
1p	1p32.3	20.41	55681039‐68297970	12616931	75	*JUN*	29.63	9.09	.16 (NS)
19	19q13.42	20.41	55995854‐56111705	115851	6	—	33.33	4.55	.033

**Figure 2:**
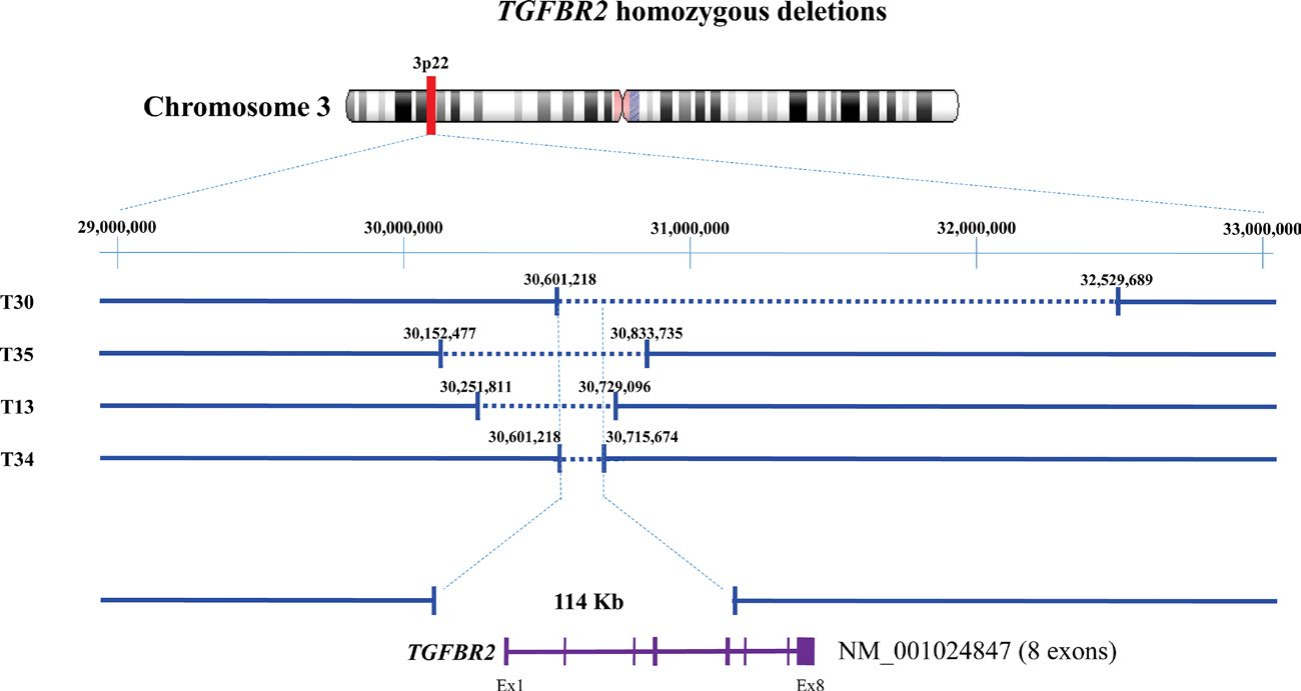
*TGFBR2* homozygous deletions

Fifty‐four homozygous deletions were identified, including 5 recurrent homozygous deletions for the loci: *LRP1B* (2q21.2; n = 2), *TGFBR2* (3p22; n = 4), *PTEN* (10q23.3; n = 2), *TRAF3* (14q32.32; n = 2), and *MACROD2* (20p12.1; n = 3) (Table 2A). The smallest common deleted region of 4 homozygous deleted tumors at 3p22 affected nucleotides 30, 601, 218‐30, 715, and 674 (deletion size of 114 456 bp) and encompassed the promoter and the first 5 exons of *TGFBR2* gene (Figure 2).

**Table 2:** Homozygous deletions (A) and focal amplifications (B) in the series of 49 ASCCs

Tumor number	Chromosome	Genomic position^a^		Size (Kb)	Candidate cancer genes	Number of additional genes
		Start	Stop			
(A) Homozygous deletions						
T42	2q	141719777	142287302	568	**LRP1B** b	0
T41	2q	141961813	142097960	136	**LRP1B**	0
T49	2q	222721345	222774000	53	—	0
T35	3p	30152477	30833735	681	**TGFBR2**	1
T13	3p	30251811	30729096	478	**TGFBR2**	0
T30	3p	30601218	32529689	1928	**TGFBR2**	8
T34	3p	30601218	30715674	114	**TGFBR2**	0
T7	3p	56942992	57108140	165	—	2
T33	3p	57389175	57614051	225	—	4
T36	3p	60431642	60504289	73	FHIT	0
T22	3q	107001161	107379667	379	—	3
T45	4q	87390704	87643400	253	PTPN13	0
T45	4q	150441915	150902655	461	—	0
T23	4q	151347901	151564275	216	—	2
T45	4q	153420602	153552364	132	FBXW7	2
T44	5q	58940595	59446730	506	—	1
T37	6p	29854870	29903186	48	—	3
T8	7q	134132030	134154953	23	—	1
T23	7q	151856130	151900145	44	MLL3	0
T14	8p	16040684	16624068	583	MSR1	0
T37	8q	107695457	107813781	118	—	2
T43	9p	6575628	6690027	114	—	1
T44	9p	8807702	10060074	1252	—	1
T44	9p	21583983	22125464	541	CDKN2A	4
T43	9q	115769754	115812331	43	—	2
T30	10p	647277	912575	265	—	3
T30	10p	4862123	5888319	1026	—	15
T31	10q	89348185	91128004	1780	**PTEN**	19
T30	10q	89625664	89722948	97	**PTEN**	0
T31	10q	101206545	101458546	252	—	3
T9	10q	103741298	103871109	130	—	3
T9	10q	124348251	124351778	4	—	1
T41	11p	10040789	10160579	120	—	1
T14	11p	19164556	19177503	13	—	1
T2	11q	85418464	85975246	557	EED	3
T7	13q	48685540	49189327	504	RB1	3
T45	13q	50747777	50876900	129	—	2
T45	13q	60342599	60715215	373	—	1
T45	13q	100793061	101042369	249	—	1
T31	14q	28339878	30047566	1708	—	3
T7	14q	103226005	103336569	111	**TRAF3**	0
T31	14q	103315491	103531760	216	**TRAF3**	2
T8	15q	60639903	60728450	89	—	0
T36	16p	6274664	6943369	669	—	1
T43	16p	21599687	21739911	140	—	3
T31	16p	32077887	33773163	1695	—	6
T4	16q	83115013	83432724	318	—	1
T31	17p	20839079	20931919	93	—	1
T45	20p	14786361	14824431	38	**MACROD2**	0
T17	20p	14808927	14916449	108	**MACROD2**	1
T41	20p	14685390	14884788	199	**MACROD2**	1
T46	Xp	50653790	50674794	21	—	1
T33	Xq	137430350	137745714	315	—	2
T49	Xp	7073279	7152984	80	—	1
(B) Focal amplifications						
T45	1p	94117825	99305935	5188	—	23
T11	1q	146633992	149243967	2609	—	4
T42	1q	150468933	150552007	83	MCL1	4
T45	1q	156955933	163053407	6097	**DDR2**	121
T11	1q	159912739	169695388	9783	**DDR2**	119
T23	1q	160549202	165142132	4592	**DDR2**	57
T45	1q	239756962	249197762	9441	—	94
T11	2q	98263510	102700794	4437	—	25
T11	2q	191215415	191612261	397	—	4
T45	3p	159711	7170996	7011	—	21
T45	3p	13466423	19384217	5918	—	39
T11	3p	82429355	83938826	1509	—	1
T27	3q	100342240	100438926	97	—	2
T11	4p	17129095	22302368	5173	—	14
T3	4q	58881742	59279688	398	—	0
T11	5p	70922229	73562716	2640	—	15
T45	5q	50399526	51613550	1214	—	1
T45	6p	5999325	7631832	1632	—	11
T45	6p	8581055	9797451	1216	—	4
T45	6p	52423397	52957234	534	—	11
T45	7q	89982243	92947957	2966	CDK6	20
T45	7q	111497007	113531594	2035	—	9
T45	7q	116428644	117701988	1273	MET	10
T18	8p	8887305	11998652	3111	—	34
T18	8p	16829810	20745858	3916	—	30
T45	8p	28165470	29132608	967	—	9
T1	8p	31705338	32599619	894	NRG1	0
T4	8p	40516566	41801388	1285	**SFRP1, NKX6‐3**	7
T17	8p	40976967	42023028	1046	**SFRP1, NKX6‐3**	7
T15	8q	51172683	51897027	724	—	1
T45	8q	127958754	128737245	778	**MYC**	2
T12	8q	128648487	128931662	283	**MYC**	1
T48	11p	2020361	2156216	0.1	IGF2	0
T42	11q	68468717	70627125	2158	**CCND1**	18
T18	11q	68777026	70256004	1479	**CCND1**	13
T13	11q	69299678	71293376	1994	**CCND1**	18
T11	12q	65432368	73724943	8293	MDM2	23
T1	13q	27690631	31048425	3358	FLT3	19
T9	15q	20416244	21933319	1517	—	6
T23	15q	64404754	64561193	156	—	4
T11	17q	52817574	53798187	981	—	6
T23	17q	69107506	71705679	2598	SOX9	10
T13	18q	30225878	33090589	2618	**MAPRE2**	8
T48	18q	31621014	32675976	1055	**MAPRE2**	3
T14	18q	58632332	61297905	2666	BCL2	12
T11	19p	1149294	1298701	149	—	9
T17	19p	13080354	13211568	131	**NFIX,LYL1**	1
T12	19p	13136304	13218303	82	**NFIX,LYL1**	1
T45	19q	37077693	40771444	3694	**AKT2**	106
T11	19q	37440672	40095021	2654	**AKT2**	57
T4	19q	37533808	39695155	2161	—	53
T3	19q	37553808	39307259	1753	—	40
T4	19q	44423396	44803072	379	—	15
T11	19q	43884667	46295324	2411	—	89
T11	19q	58419791	59082951	663	—	33
T11	21q	20872889	23530026	2657	—	2
T21	21q	29671040	31528208	1857	—	12
T45	21q	32496947	37246707	4749	—	16
T45	21q	37818180	39354904	1536	**DYRK1A, KCNJ6**	7
T11	21q	38668355	39992546	1324	**DYRK1A, KCNJ6**	5
T11	22q	16915658	17768070	852	—	11
T2	Xq	107275387	108021257	745	—	2
T40	Xq	148845624	149029121	183	—	5

a Human GRCh37/hg19.

b Recurrent altered genes in bold characters.

### 3.4 Genomic gain and focal amplification

The most significantly frequent minimal regions of gain with a frequency greater than 25% were located at loci 1p36.33 (37%), 1q21.1 (29%), 3q26.32 (57%), 5p15.33 (29%), 8q24.3 (39%), 9q34.3 (33%), 16p13.3 (41%), and 19p13.3 (27%) (Table 1B; Figure 1A). The most common frequent minimal region of gain in 57% of ASCCs encompassed the 3q26.32 region containing *PIK3CA* and *TERC* (Figure S3). *PIK3CA (*but not *TERC)* mRNA was significantly overexpressed in the ASCCs with 3q26.32 gains, compared to diploid tumors (*P *=* *.013; Figure S3), suggesting that *PIK3CA* is the likely target of this gain event in this chromosomal region. Other common regions of genomic gain containing known oncogenes were located at 9q34.3 (*NOTCH1*) and 19p13.3 (*FGFR2*) (Table 1B). Sixty‐three focal amplifications were identified, including 8 recurrent focal amplifications for the loci: *DDR2* (1q23.3; n = 3), *SFRP1* and *NKX6‐3* (8p11.21; n = 2), *MYC* (8q24.21; n = 2), *CCND1* (11q13; n = 3), *MAPRE2* (18q12.1; n = 2), *NFIX, LYL1* (19p13.2; n = 2), *AKT2* (19q13.2; n = 2), and *DYRK1A* and *KCNJ6* (21q22.13; n = 2). It is noteworthy that 7 of the amplified genes in focal amplifications (ie, *DDR2, CDK6, MET, IGF2, MDM2, FLT3,* and *AKT2*) are targets for specific therapies (Table 2B).

### 3.5 Comparative genomic analysis of treatment‐naive and recurrent tumor samples

As described in the literature, accumulation of gains/amplification and/or loss in the genome can generate a pattern of chromosomal alterations, which could specifically contribute to cancer progression. Based on these elements, we investigated whether such patterns of chromosomal abnormalities could be preferentially observed in the 22 recurrent tumors compared to the 27 treatment‐naive tumors. The global load of genomic alterations was similar between relapsed and treatment‐naive tumors, including the number of distinct CGH segments identified [median = 150 (range: 68‐550) and median = 126 (range: 95‐386), respectively] and the fraction of altered genome [median = 15.84 (range: 1.81‐51.94) and median = 18.74 (range: 0.96‐38.03), respectively] (Table S4). Several individual genomic alterations (Figure 1B,C) were more frequently (but not statistically significantly) observed in the recurrent tumor group compared to the treatment‐naive tumor group including, for example, heterozygous deletions at 2q35 (36% vs 19%) or gains at 5p15.33 (36% vs 22%) (Table 1A,B). More surprisingly, several individual genomic alterations were statistically more frequently observed in the treatment‐naive tumor group as compared to the recurrence tumor group, in particular heterozygous deletions at 11q14.2 (48% vs 9%; *P *=* *.003) or gains at 19q13.42 (33% vs 5%; *P *=* *.03) (Table 1A,1B). It is noteworthy that the 11q14.2 deleted region contains the well‐known tumor suppressor gene *EED* that also shows a homozygous deletion (Table 2A).

### 3.6 Mutations and CNA involved in key cell signaling pathways in ASCC

Data concerning *PIK3CA, KRAS,* and *TP53* mutations and the most frequent CNA were combined to characterize genomic alterations in the main signaling pathways altered in human cancers. Nineteen of the 49 tumors (38.8%) harbored gene mutations: *PIK3CA* mutation in 16 (32.6%) cases, *KRAS* mutation in 2 (4.1%) cases, and *TP53* mutation in 2 (4.1%) cases. One tumor harbored both a *KRAS* mutation and a *TP53* mutation. All tumors were wild‐type for *BRAF* gene. The distribution of molecular, biological, pathological, and clinical parameters was similar between treatment‐naive tumors and recurrences, except for *PIK3CA (*or *KRAS)* mutations, which were significantly more frequent in recurrences (*P *=* *.02) (Table S3).

Among CNAs, only focal amplifications and homozygous deletions were integrated in signaling pathways (Table 3). The most frequently altered pathway in the 49 tumors was the PI3K/Akt/mTOR pathway, which was altered in 22 of the 49 tumors (44.9%). PI3K/Akt/mTOR pathway alterations included activating mutations of *PIK3CA*, homozygous deletion of *PTEN,* and focal amplifications of *IGF2* and *AKT2,* and all of these somatic events were mutually exclusive. Interestingly, the PI3K/Akt/mTOR pathway was altered significantly more frequently in recurrences than in treatment‐naive tumors (64% vs 30%; *P *=* *.017) (Table 3). It is noteworthy that the RAS/MAPK signaling pathway was rarely altered (2 of the 49 tumors; 4.1%).

**Table 3:**
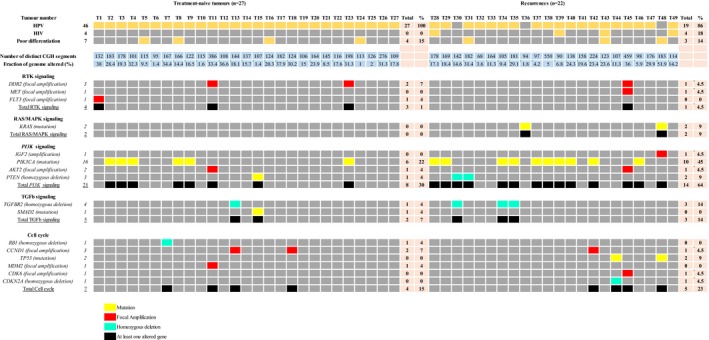
Common genetic alterations in signaling pathways in the series of 49 ASCCs

### 3.7 Correlation between genomic indices and clinicopathological features and prognostic value

As this retrospective cohort of 49 ASCC patients comprised tumor samples with heterogeneous sites and treatment status, the study population was divided into 2 groups of patients (treatment‐naive tumors and recurrences) to study the association between the 2 genomic indices (number of distinct CGH segments identified and fraction of genome altered) and the patients’ clinicopathological characteristics, and the impact of these 2 indices on OS.

The first group of treatment‐naive tumors from 27 ASCC patients treated by first‐line exclusive RT/CRT (Table S3) had a median follow‐up of 44.6 months (range: 13.9‐169 months). The overall recurrence rate was 29.6% (n = 8 of 27). No correlation was observed between the 2 genomic indices and OS (data not shown).

The second group of 22 recurrent tumor samples (20 anal recurrences treated by APR and 2 metastases) from patients with ASCC who experienced recurrence after first‐line RT or CRT (Table S3) had a median follow‐up of 46.7 months (range: 9.8‐278 months). The overall mortality rate after recurrence was 63.4% (n = 14 of 22).

**Figure 3:**
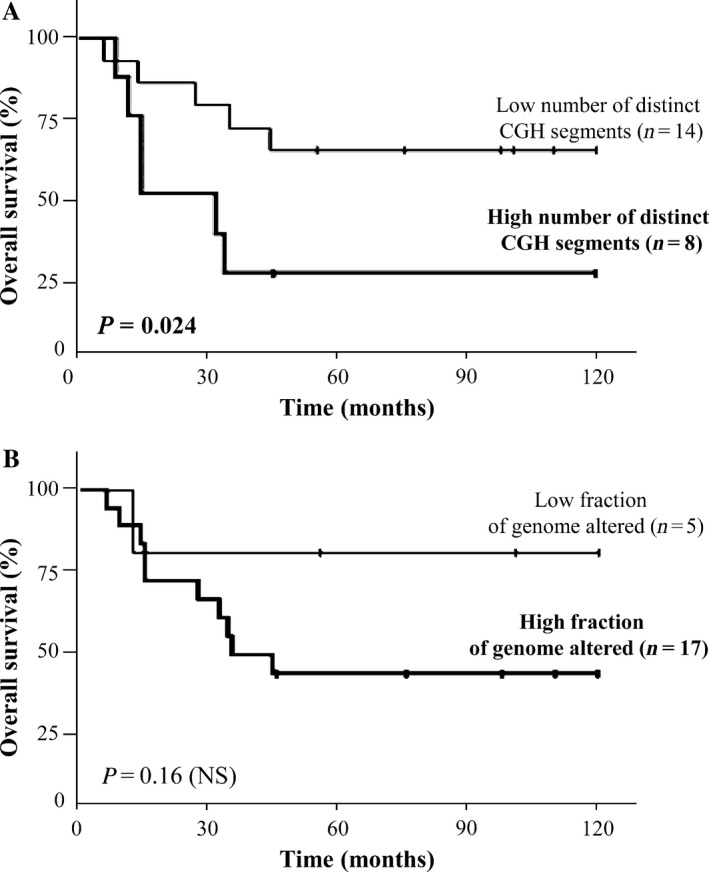
Overall survival in the 22 ASCC patients with recurrent tumors depending on the “distinct CGH segments” status (A) and the “fraction of genome altered” status (B)

Long‐rank test demonstrated a significant correlation between poor OS and a large number of distinct CGH segments in recurrent tumors (*P *=* *.024) (Figure 3A), and a trend toward significance for a high fraction of the genome altered in treatment‐naive tumors (*P *=* *.16) (Figure 3B). No correlation was observed between the number of distinct CGH segments and clinicopathological characteristics in the group of 22 recurrent tumors (Table S5) or in the group of 29 treatment‐naive tumors (data not shown).

## 4 DISCUSSION

ASCC is considered to be a highly radiosensitive tumor, but 20% of patients fail to respond to CRT. No predictive markers of response to radiation‐based therapy have been prospectively validated. Moreover, in patients who develop recurrence, APR is the treatment of choice, but no prognostic factors have been identified and no adjuvant therapy has been recommended. More accurate genomic characterization of anal carcinogenesis is crucial to improve the medical care of patients with ASCC by identifying new therapeutic targets or prognostic biomarkers. In this context, we conducted a large array comparative genomic hybridization analysis in treatment‐naive and recurrent ASCC.

Despite previous exposure to ionizing radiation and DNA‐damaging cytotoxic chemotherapy, the global load of genomic alterations was high but similar between treatment‐naive tumors and recurrences, in line with the mutational burden described in whole‐exome analysis of ASCC^17^ and in other types of carcinoma.^18^, ^19^ Surprisingly, several individual genomic alterations were observed more frequently in the group of treatment‐naive tumors.

A significant correlation was demonstrated between genomic index and OS in the group of recurrent tumors (not observed in the group of treatment‐naive tumors), with a high number of distinct CGH segments associated with poor prognosis. Due to the little size of our cohort, this correlation needs to be confirmed in a larger prospective randomized study.

Several recurrent minimal heterozygous deleted regions were identified in this study. It is noted that our methodology (CGH microarray but not SNP array) did not allow to estimate copy number neutral loss of heterozygosity (LOH). The most common frequent minimal region of deletion encompassed the 11q22.3 region, containing *ATM*. Five recurrent homozygous deletions were identified in the *TGFBR2* (8%), *MACROD2* (6%), *PTEN* (4%), *LRP1B* (4%), and *TRAF3* (4%) loci. Homozygous deletions of *TGFBR2*,* LRP1B,* and *TRAF3* genes have never been previously reported in ASCC. The *TGFBR2* gene is involved in homeostasis of many tissues via the TGFβ signaling pathway. It encodes a tyrosine kinase receptor that is involved in cell proliferation, epithelial‐mesenchymal transition, and apoptosis. Bi‐allelic inactivation of TGFBR2 using a keratin 14 promoter in mice leads to spontaneous genital and anal SCC.^20^ Homozygous deletion of *TGFBR2* has been reported in gastric and pancreatic cancer,^21^, ^22^ and alteration of *TGFBR2* expression is associated with poor prognosis in several cancers.^23^, ^24^ The *LRP1B* gene encodes a member of the LDL receptor family of lipoprotein receptors that is involved in cholesterol metabolism and atherosclerotic lesion formation. Homozygous deletion of *LRP1B* has been reported in multiple malignancies, namely esophageal cancer, glioblastoma, and cervical cancer.^25^, ^26^, ^27^ *LRP1B* gene has been recently identified as the integration site for HPV in cervical and oropharyngeal cancers.^27^, ^28^ The *TRAF3* gene encodes a cytoplasmic adaptor protein, with E3 ligase activity, which is involved in the signaling of a variety of adaptive and innate immune receptors as well as cytokine receptors. In particular, homozygous deletions of the *TRAF3* gene have been detected in hematopoietic malignancies, such as multiple myeloma, non‐Hodgkin lymphoma, and B‐cell chronic lymphocytic leukemia.^29^, ^30^ *TRAF3* has recently been shown to be downregulated by HPV via upregulation of *UCHL1* with suppression of the innate immune response in keratinocytes.^31^

Several recurrent minimal regions of gain were identified. The most common frequent minimal region of gain, observed in 66% of ASCCs, encompassed the 3q26.32 region containing *PIK3CA* and *TERC*. Moreover, the RNA results identified *PIK3CA* (and not *TERC*) as the driver gene of this 3q26.32 region of gain. The PI3K/Akt/mTOR pathway has been often identified in previous ASCC sequencing studies with PIK3CA mutations in 20% to 30% of ASCC.^32^, ^33^ Other common regions of genomic gain that contain known oncogenes, with potential therapeutic interest, were located at 9q34.3 (*NOTCH1*) and 19p13.3 (*FGFR2*).

Several recurrent focal amplifications of known oncogenes with possible therapeutic implications were also identified: *AKT2* (8%)*, DDR2* (6%), and *IGF2* (4%), which are known to be targets for specific therapies and which could be used as novel agents in the treatment of ASCC. The *AKT2* gene is a partner of the PI3K/Akt/mTOR pathway and is known to be amplified in HPV‐associated squamous cell cancers.^34^ Four of the other focal amplifications (ie, *CDK6, MET, MDM2, and FLT3*) are also targets for specific therapies.

Considering the high prevalence of HPV infection in ASCC (approximately 95%) and its well‐established role in the first steps of anal carcinogenesis, it seems difficult to distinguish signaling pathway changes caused by genetic mutations from those caused by HPV. However, *TP53* mutations have been reported more frequently in the rare HPV‐negative cases of ASCC^10^, ^33^, ^35^, ^36^ and could therefore be involved in another pathway of anal carcinogenesis.

In conclusion, this study represents the largest array comparative genomic hybridization analysis in treatment‐naive and recurrent ASCC. The results of this study further our knowledge of the genetic landscape of ASCC and highlight the crucial role of biological and molecular characterization of rare diseases for the development of new treatments. This study identifies new tumor suppressor genes, *LRP1B* and *TRAF3*, with possible interactions with HPV and confirmed the role of *TGFBR2* and *PTEN* in ASCC carcinogenesis. We confirm the major role of activation of the PI3K/Akt/mTOR pathway in ASCC carcinogenesis (45% of tumors samples) as previously described,^32^, ^33^ and in particular in recurrences, in which activation of this pathway was present in 66% of tumor samples. We also suggest several druggable target genes of this signaling pathway, such as *IGF2*,* PIK3CA,* and *AKT2*.

Clinical studies based on prospective cohorts of patients with ASCC need to be conducted in order to demonstrate the antitumor efficacy of new targeting agents in light of the molecular alterations identified in the present study.

## CONFLICT OF INTERESTS

The authors have declared that no competing interests exist.
