# Supplementary material for: Array comparative genomic hybridization identifies high level of PI3K/Akt/mTOR pathway alterations in anal cancer recurrences
Source: Cancer Med. 2018 May 26;7(7):3213–25. doi: 10.1002/cam4.1533 (PMC6051172; doi:10.1002/cam4.1533)
Supplement: Supplementary file 1 [file CAM4-7-3213-s001.pptx]

## Slide 1
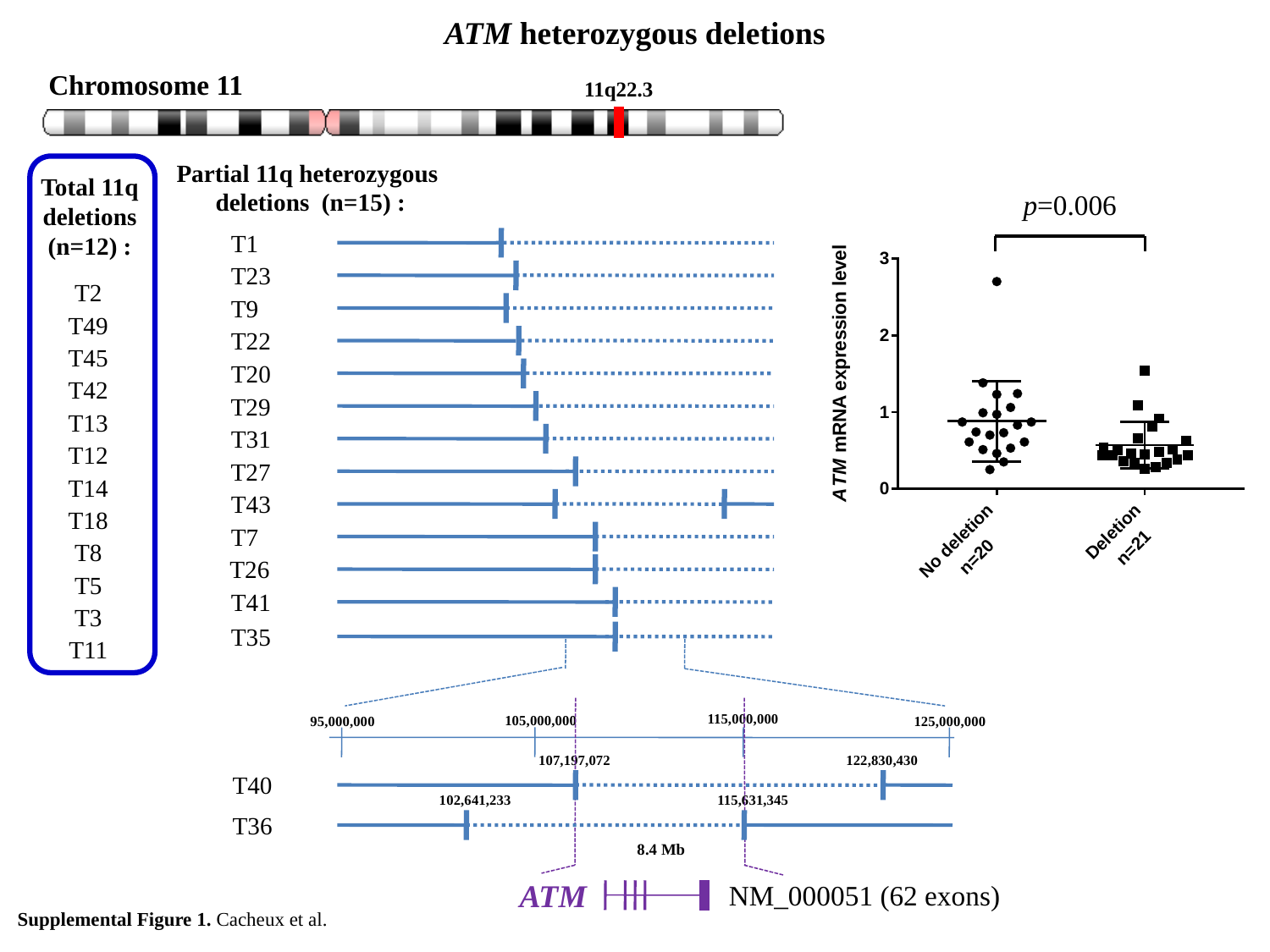

ATM heterozygous deletions
Chromosome 11
11q22.3
Partial 11q heterozygous
 deletions (n=15) :
Total 11q deletions
(n=12) :
p=0.006
T1
T23
T2
T9
T49
T22
T45
T20
T42
T29
T13
T31
T12
T27
T14
T43
T18
T7
T8
T26
T5
T41
T3
T35
T11
115,000,000
105,000,000
95,000,000
125,000,000
107,197,072
122,830,430
T40
102,641,233
115,631,345
T36
8.4 Mb
ATM
NM_000051 (62 exons)
Supplemental Figure 1. Cacheux et al.
