# Supplementary material for: Array comparative genomic hybridization identifies high level of PI3K/Akt/mTOR pathway alterations in anal cancer recurrences
Source: Cancer Med. 2018 May 26;7(7):3213–25. doi: 10.1002/cam4.1533 (PMC6051172; doi:10.1002/cam4.1533)
Supplement: Supplementary file 2 [file CAM4-7-3213-s002.pptx]

## Slide 1
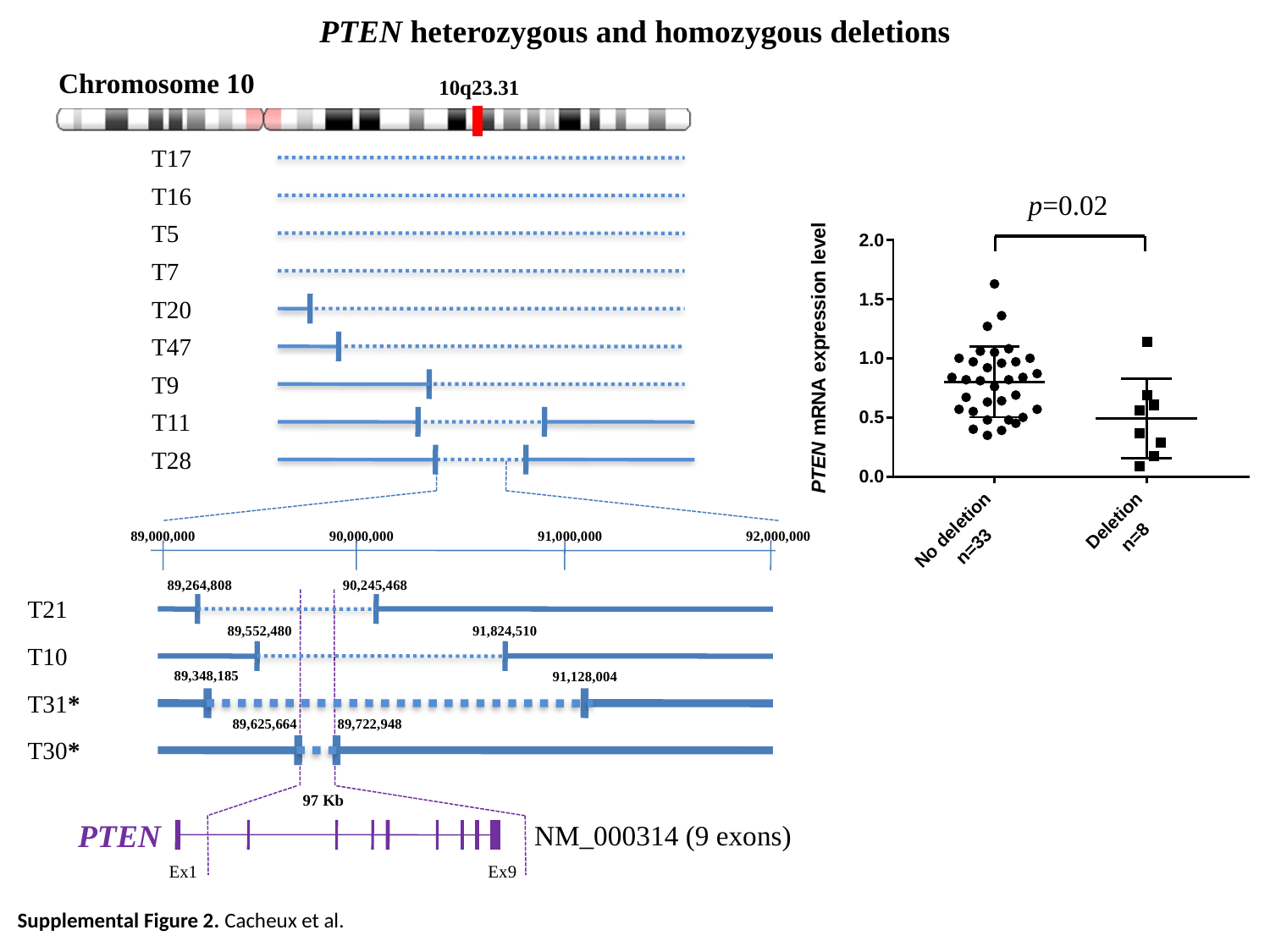

PTEN heterozygous and homozygous deletions
Chromosome 10
10q23.31
T17
T16
p=0.02
T5
T7
T20
T47
T9
T11
T28
89,000,000
90,000,000
91,000,000
92,000,000
89,264,808
90,245,468
T21
89,552,480
91,824,510
T10
89,348,185
91,128,004
T31*
89,625,664
89,722,948
T30*
97 Kb
PTEN
NM_000314 (9 exons)
Ex1
Ex9
Supplemental Figure 2. Cacheux et al.
