# Supplementary material for: Array comparative genomic hybridization identifies high level of PI3K/Akt/mTOR pathway alterations in anal cancer recurrences
Source: Cancer Med. 2018 May 26;7(7):3213–25. doi: 10.1002/cam4.1533 (PMC6051172; doi:10.1002/cam4.1533)
Supplement: Supplementary file 3 [file CAM4-7-3213-s003.pptx]

## Slide 1
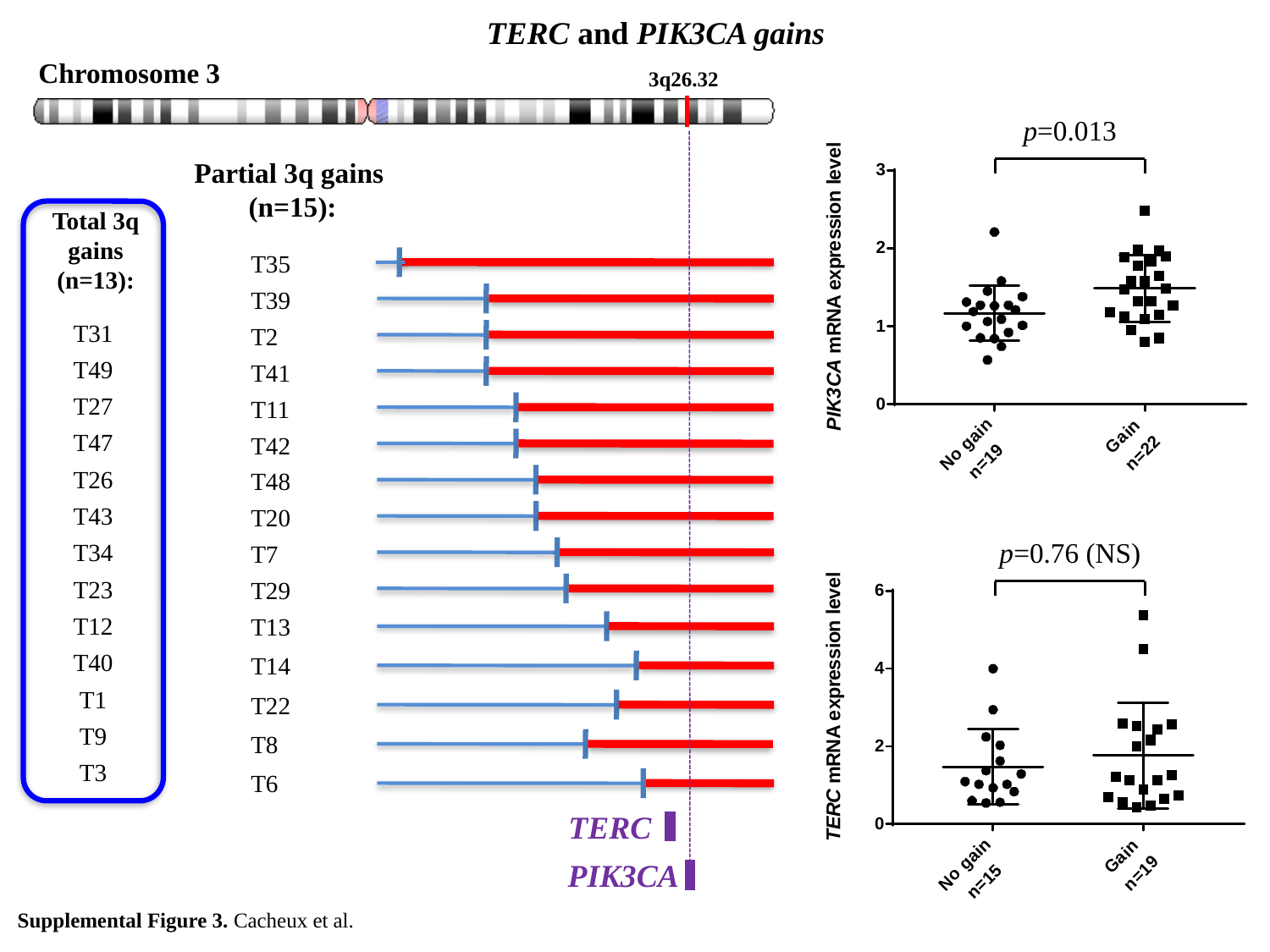

TERC and PIK3CA gains
Chromosome 3
3q26.32
p=0.013
Partial 3q gains
(n=15):
Total 3q
gains
(n=13):
T35
T39
T31
T2
T49
T41
T27
T11
T47
T42
T26
T48
T43
T20
p=0.76 (NS)
T34
T7
T23
T29
T12
T13
T40
T14
T1
T22
T9
T8
T3
T6
TERC
PIK3CA
Supplemental Figure 3. Cacheux et al.
