# Supplementary material for: Array comparative genomic hybridization identifies high level of PI3K/Akt/mTOR pathway alterations in anal cancer recurrences
Source: Cancer Med. 2018 May 26;7(7):3213–25. doi: 10.1002/cam4.1533 (PMC6051172; doi:10.1002/cam4.1533)
Supplement: Supplementary file 5 [file CAM4-7-3213-s005.doc]

**Supplementary Table 2.** Primers used for RT-qPCR for the selected genes.

| **Gene** | **Upper sequence** | **Lower sequence** | **Amplicon size (pb)** |
| --- | --- | --- | --- |
| ***PTEN*** | 5' GTG GCG GAA CTT GCA ATC CT 3' | 5' ATG AAC TTG TCT TCC CGT CGT GT 3' | 97 |
| ***ATM*** | 5' CCA GCT GTG CAG CGA ACA AT 3' | 5' TCT AAG CAC GTT TCT GCT AAC CAG T 3' | 92 |
| ***PIK3CA*** | 5' CCT GAT CTT CCT CGT GCT GCT C 3' | 5' ATG CCA ATG GAC AGT GTT CCT CTT 3' | 92 |
| ***TERC*** | 5' CCG CCT TCC ACC GTT CAT TC 3' | 5' AAC GGG CCA GCA GCT GAC A 3' | 55 |
| ***TBP*** | 5’ TGC ACA GGA GCC AAG AGT GAA 3’ | 5’ CAC ATC ACA GCT CCC CAC CA 3’ | 132 |
